# Supplementary material for: Enhancing prediction accuracy of coronary artery disease through machine learning-driven genomic variant selection
Source: J Transl Med. 2024 Apr 16;22:356. doi: 10.1186/s12967-024-05090-1 (PMC11020205; doi:10.1186/s12967-024-05090-1)
Supplement: Supplementary file 1 — Additional file 1: Table S1. List of CAD-related phenotypes that were used to select CAD cases within the UK Biobank cohort over a 12 year follow-up period. Table S2. Association statistics between the identified loci and CAD in various cohort-based studies. Table S3. List of other major CVD GWAS associations by using the selected ML-driven loci. Figure S1. Summary of GWAS QC and initial pre-processing. UKBB raw imputation genotypes consisted of 97 million markers, that were subjected to QCs steps shown here. Figure S2. Principal component analysis applied to genotype data to uncover population structure and use the PCs as covariates. A Variance explained by the first 20 PCs. B Scatter plots that display the variance explained by the first 6 PCs. Different colors are associated to different ethnic groups in order to visually show the population structure among the individual of the UK Biobank cohort. Figure S3. SHAP summary plot. This plot shows the SHAP values for each feature and observation (dots). Each dot has three characteristics: vertical location shows what feature it is depicting; color shows whether that feature was high or low for that row of the dataset; horizontal location shows whether the effect of that value caused a higher or lower prediction. Figure S4. Correlation-based analysis comparing risk factors, principal components and ethnicity. (A) Standard correlation analysis showing associations between risk factors and PCs and ethnic group and PCs. (B-C) Variance inflation factor (VIF) to measure of the amount of multicollinearity in a set of multiple regression variables. (B) VIF results before (B) and after removing (C) cholesterol and ethnicity. [file 12967_2024_5090_MOESM1_ESM.pdf]

A computational framework using machine learning-driven feature selection for predictions of Coronary Artery Disease

Z. Alireza,[1] M. Maleeha, M. Kaikkonen, and V. Fortino[1*]

[1] Institute of Biomedicine, University of Eastern Finland, 70210 Kuopio, Finland

* To whom correspondence should be addressed. Tel: +358 50 326 6148;
Email: vittorio.fortino@uef.fi

Supplementary Figures and Tables

Supplementary Table 1 –List of CAD-related phenotypes that were used to select CAD cases within the UK Biobank cohort over a 12-year follow-up period.

	Code	Meaning
ICD - 9	410	Acute myocardial infarction
	4109	Acute myocardial infarction
	411	Other acute and subacute forms of ischaemic heart disease
	4119	Other acute and subacute forms of ischaemic heart disease
	412	Old myocardial infarction
	4129	Old myocardial infarction
	4299	Heart disease and complications, unspecified
ICD - 10	I21	Acute myocardial infarction
	I210	Acute transmural myocardial infarction of anterior wall
	I211	Acute transmural myocardial infarction of inferior wall
	I212	Acute transmural myocardial infarction of other sites
	I213	Acute transmural myocardial infarction of unspecified site
	I214	Acute subendocardial myocardial infarction
	I219	Acute myocardial infarction, unspecified
	I21X	Presumed acute myicardial infaction (unconfirmed)
	I22	Subsequent myocardial infarction
	I220	Subsequent myocardial infarction of anterior wall
	I221	Subsequent myocardial infarction of inferior wall
	I228	Subsequent myocardial infarction of other sites
	I229	Subsequent myocardial infarction of unspecified site
	I23	Certain current complications following acute myocardial infarction
	I230	Haemopericardium as current complication following acute myocardial infarction
	I231	Atrial septal defect as current complication following acute myocardial infarction
	I232	Ventricular septal defect as current complication following acute myocardial infarction
	I233	Rupture of cardiac wall without haemopericardium as current complication following acute myocardial infarction
	I234	Rupture of chordae tendineae as current complication following acute myocardial infarction
	I235	Rupture of papillary muscle as current complication following acute myocardial infarction
	I236	Thrombosis of atrium, auricular appendage and ventricle as current complications following acute myocardial infarction
	I238	Other current complications following acute myocardial infarction
	I241	Dressler's syndrome
	I252	Old myocardial infarction
OPCS - 4	K401	Saphenous vein graft replacement of one coronary artery
	K402	Saphenous vein graft replacement of two coronary arteries
	K403	Saphenous vein graft replacement of three coronary arteries
	K404	Saphenous vein graft replacement of four or more coronary arteries
	K411	Autograft replacement of one coronary artery NEC
	K412	Autograft replacement of two coronary arteries NEC
	K413	Autograft replacement of three coronary arteries NEC
	K414	Autograft replacement of four or more coronary arteries NEC
	K451	Double anastomosis of mammary arteries to coronary arteries
	K452	Double anastomosis of thoracic arteries to coronary arteries NEC
	K453	Anastomosis of mammary artery to left anterior descending coronary artery
	K454	Anastomosis of mammary artery to coronary artery NEC
	K455	Anastomosis of thoracic artery to coronary artery NEC
	K491	Percutaneous transluminal balloon angioplasty of one coronary artery
	K492	Percutaneous transluminal balloon angioplasty of multiple coronary arteries
	K498	Other specified transluminal balloon angioplasty of coronary artery
	K499	Unspecified transluminal balloon angioplasty of coronary artery
	K502	Percutaneous transluminal coronary thrombolysis using streptokinase
	K751	Percutaneous transluminal balloon angioplasty and insertion of 1-2 drug-eluting stents into coronary artery
	K752	Percutaneous transluminal balloon angioplasty and insertion of 3 or more drug-eluting stents into coronary artery
	K753	Percutaneous transluminal balloon angioplasty and insertion of 1-2 stents into coronary artery
	K754	Percutaneous transluminal balloon angioplasty and insertion of 3 or more stents into coronary artery NEC
	K758	Other specified percutaneous transluminal balloon angioplasty and insertion of stent into coronary artery
	K759	Unspecified percutaneous transluminal balloon angioplasty and insertion of stent into coronary artery

Supplementary Table 2 – Association statistics between the identified loci and CAD in various cohort-based studies.

			UKBB		CVD hugeamp (03/2023)		Finngen r8 (I9_CORATHER)		Aragam 2022		CARDIoGRAMplusC4D 2022 CAD GWAS: European ancestry		
variant	Ref/Alt	effect allele	p-value	beta/odds ratio	p-value	beta/odds ratio	p-value	beta/odds ratio	p-value	beta/odds ratio	p-value	beta/odds ratio	Oth
rs9644860	C/T	T	.	.	2,40E-239	0.17/1.19	3,20E-119	0.20/1.22	2,40E-239	0.17/1.18	4,62E-182	0.17/1.19	Myocard
rs7168951	A/C	C	8,70E-12	0.08/1.08	4,10E-18	0.04/1.04	7,30E-05	0.03/1.03	3,90E-22	0.05/1.05	2,05E-15	0.04/1.04	Myocard
rs646776	C/T	T	2,10E-25	0.14/1.15	1,32E-65	0.10/1.10	2,80E-21	0.10/1.11	3,07E-59	0.09/1.10	1,84E-53	0.09/1.10	LDL chol
rs1412740	A/G	G	7,30E-10	-0.07/0.93	1,40E-30	-0.05/0.95	7,20E-08	-0.05/0.95	1,26E-26	-0.05/0.95	1,28E-28	-0.05/0.95	Myocard
rs55730499	C/T	T	9,70E-73	0.37/1.45	1,66E-198	0.31/1.36	4,40E-47	0.28/1.32	8,26E-186	0.30/1.35	8,26E-186	0.30/1.35	Lp(a), to
rs77140532	A/G	G	1,70E-15	-0.14/0.87	1,56E-38	-0.10/0.90	5,70E-20	-0.13/0.88	2,37E-39	-0.10/0.90	1,86E-37	-0.10/0.90	LDL chol

Supplementary Table 3 – List of other major CVD GWAS associations by using the selected ML-driven loci.

variant	Other major CVD GWAS associations	Main predicted target gene	References to gene mechanism
rs9644860	Myocardial infarction	*CDKN2A/B*	PMID: 30460243
rs7168951	Myocardial infarction	*FES/FURIN*	PMID: 36321446, 32067586
rs646776	LDL cholesterol, ApoB, total cholesterol	*SORT1*	PMID: 25702058
rs1412740	Myocardial infarction	*PHACTR1/EDN1*	PMID: 35387481, 28753427
rs55730499	Lp(a), total cholesterol, LDL cholesterol, ApoB,	*LPA*	PMID: 36036785
rs77140532	LDL cholesterol	*LDLR*	PMID: 28444290

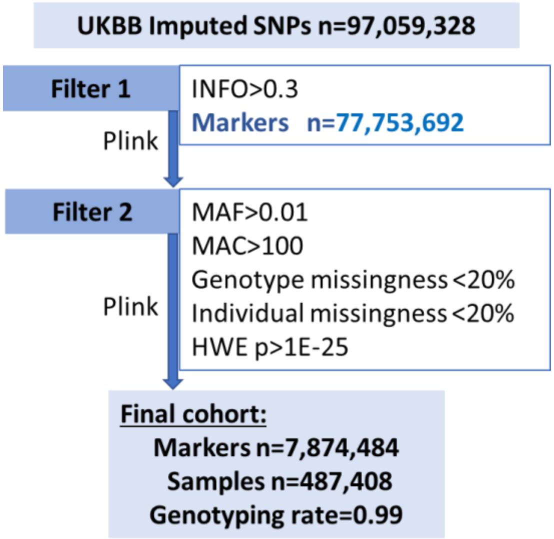

Supplementary Figure 1. Summary of GWAS QC and initial pre-processing. UKBB raw imputation genotypes consisted of 97 million markers, that were subjected to QCs steps shown here.

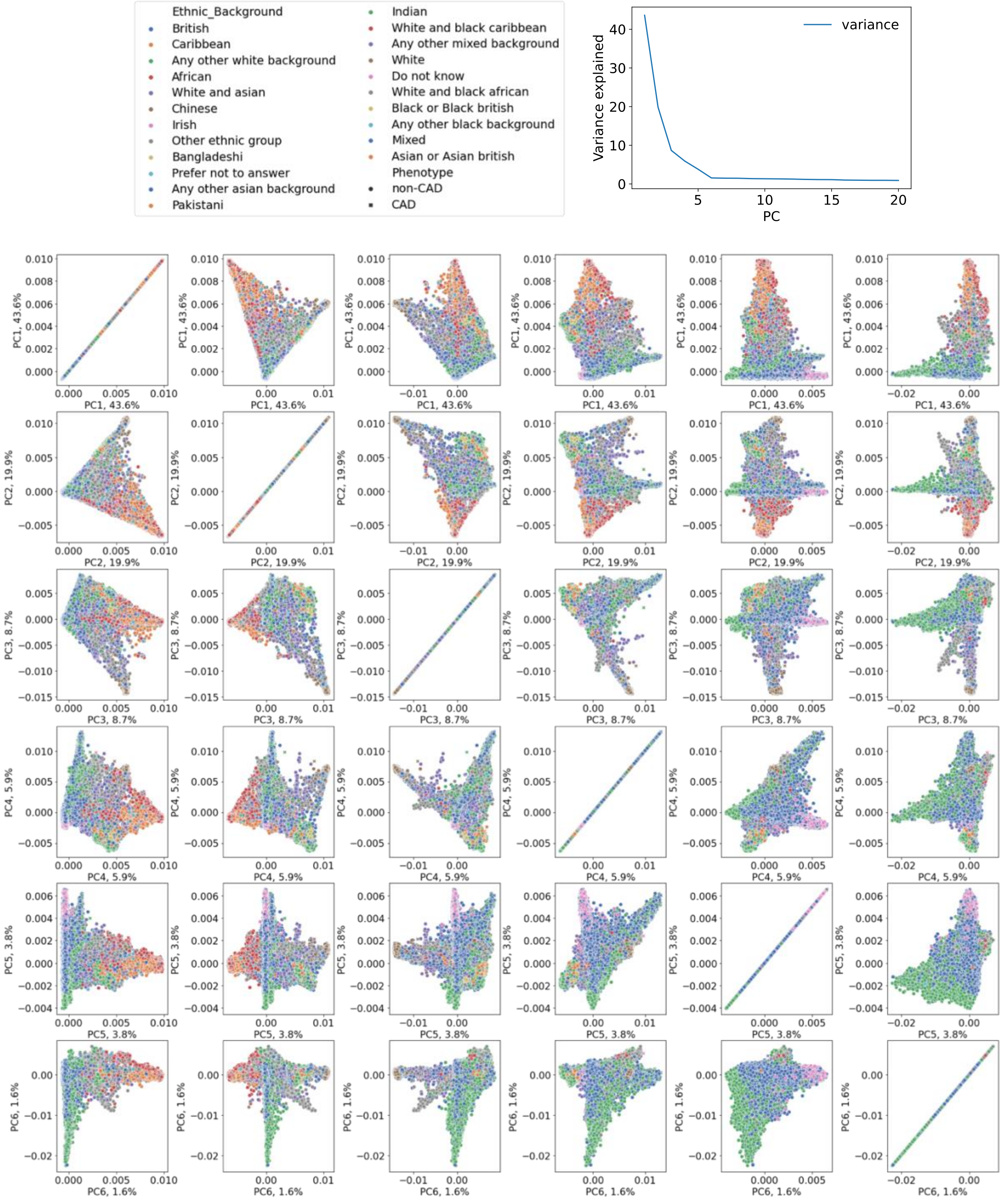

Supplementary Figure 2 - Principal component analysis applied to genotype data to uncover population structure and use the PCs as covariates. (A) Variance explained by the first 20 PCs. (B) Scatter plots that display the variance explained by the first 6 PCs. Different colors are associated to different ethnic groups in order to visually show the population structure among the individual of the UK Biobank cohort.

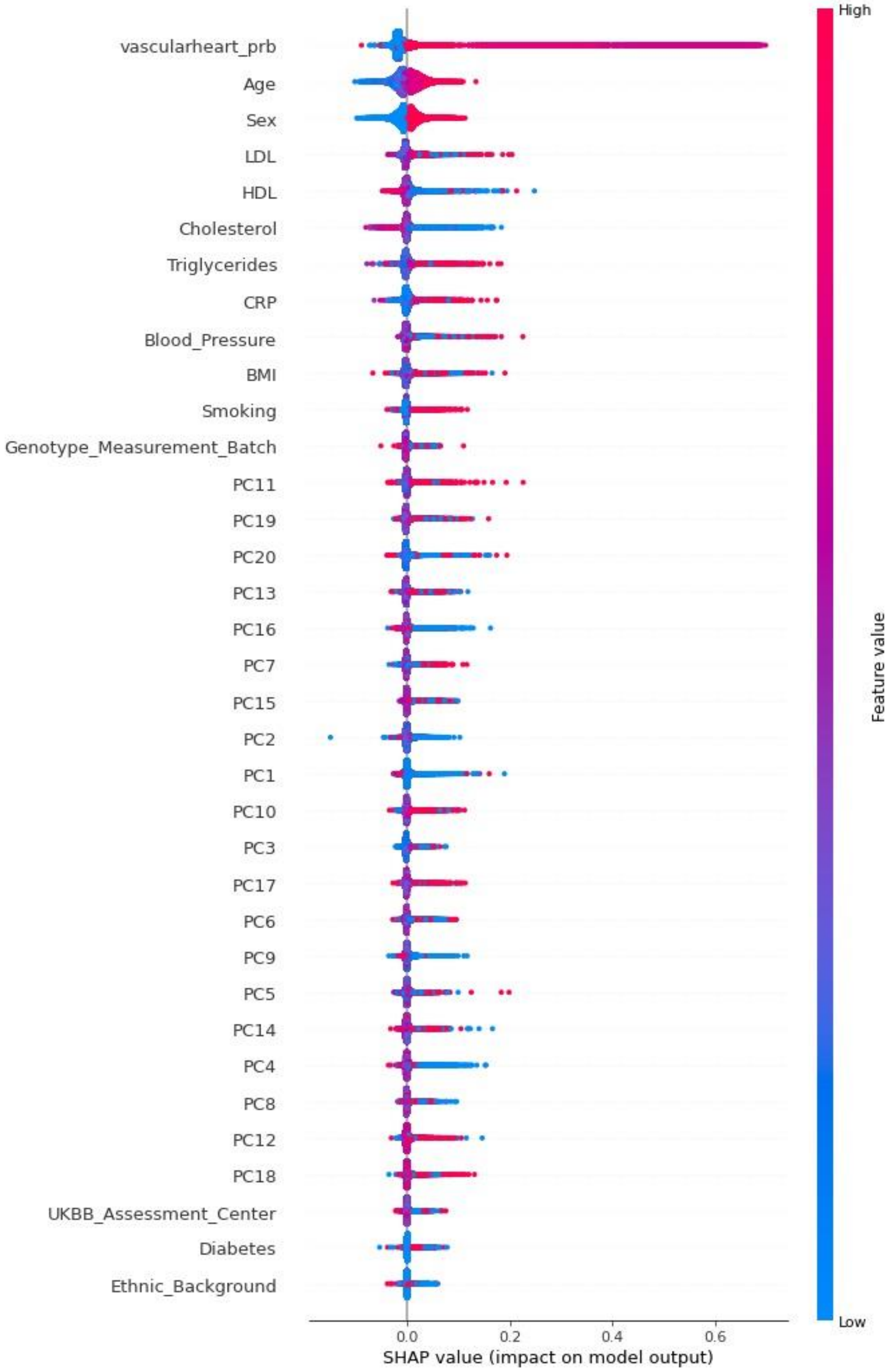

Supplementary Figure 3 – SHAP summary plot. This plot shows the SHAP values for each feature and observation (dots). Each dot has three characteristics: vertical location shows what feature it is depicting; color shows whether that feature was high or low for that row of the dataset; horizontal location shows whether the effect of that value caused a higher or lower prediction.

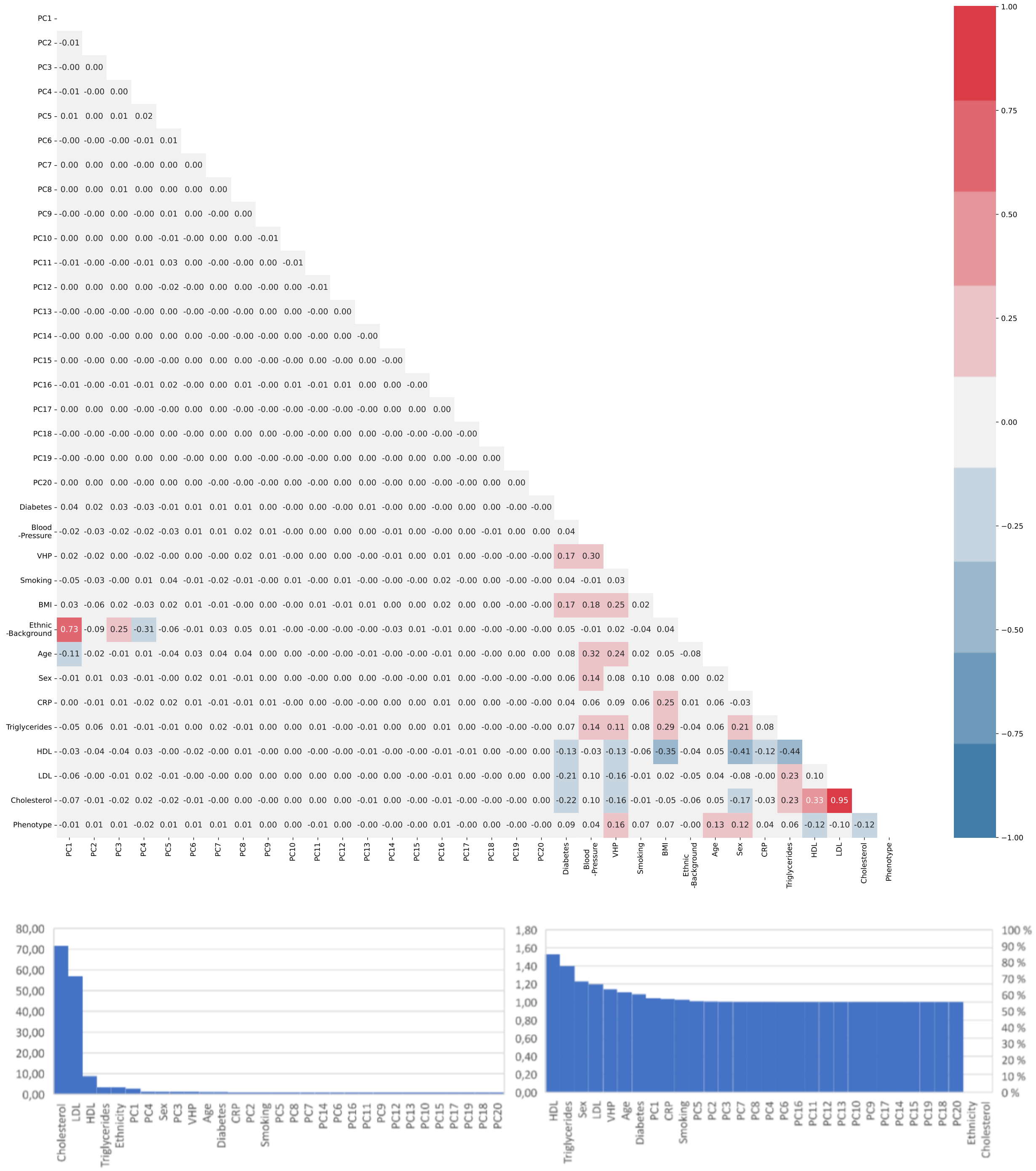

Supplementary Figure 4 – Correlation-based analysis comparing risk factors, principal components and ethnicity. (A) Standard correlation analysis showing associations between risk factors and PCs and ethnic group and PCs. (B-C) Variance inflation factor (VIF) to measure of the amount of multicollinearity in a set of multiple regression variables. (B) VIF results before (B) and after removing (C) cholesterol and ethnicity.
